# Identification of single nucleotide variants in the Moroccan population by whole-genome sequencing

**DOI:** 10.1186/s12863-020-00917-4

**Published:** 2020-09-21

**Authors:** Lucy Crooks, Johnathan Cooper-Knock, Paul R. Heath, Ahmed Bouhouche, Mostafa Elfahime, Mimoun Azzouz, Youssef Bakri, Mohammed Adnaoui, Azeddine Ibrahimi, Saaïd Amzazi, Rachid Tazi-Ahnini

**Affiliations:** 1 Centre for Mass Spectrometry Imaging, Biomolecular Sciences Research Centre, Sheffield Hallam University, Howard Street, Sheffield,, S1 1WB UK; 2 Neuroscience (SITraN), Faculty of Medicine, Dentistry and Health, University of Sheffield, Sheffield, UK; 3 Neurology and Neurogenetics, Genomic of Human Pathlogies Center, Medical School and Pharmacy, Mohammed-V University, Rabat, Morocco; 4 CNRST, Mohammed-V University, Rabat, Morocco; 5 Laboratory of human pathologies Biology BioPatH-Faculty of science-Center of human pathologies Genomics, GenoPatH- Faculty of Medicine- Mohammed V University in Rabat, Rabat, Morocco; 6 Medical School and Pharmacy, Mohammed V University, Rabat, Morocco; 7 Lab (MedBiotech), Medical School and Pharmacy, Mohammed V University, Rabat, Morocco; 8 Infection, Immunity and Cardiovascular Disease, Faculty of Medicine, Dentistry and Health, University of Sheffield, Sheffield, S10 2RX UK

**Keywords:** Whole genome sequencing, Population genomics, Africa, SNVs

## Abstract

**Background:**

Large-scale human sequencing projects have described around a hundred-million single nucleotide variants (SNVs). These studies have predominately involved individuals with European ancestry despite the fact that genetic diversity is expected to be highest in Africa where *Homo sapiens* evolved and has maintained a large population for the longest time. The African Genome Variation Project examined several African populations but these were all located south of the Sahara. Morocco is on the northwest coast of Africa and mostly lies north of the Sahara, which makes it very attractive for studying genetic diversity. The ancestry of present-day Moroccans is unknown and may be substantially different from Africans found South of the Sahara desert, Recent genomic data of Taforalt individuals in Eastern Morocco revealed 15,000-year-old modern humans and suggested that North African individuals may be genetically distinct from previously studied African populations.

**Results:**

We present SNVs discovered by whole genome sequencing (WGS) of three Moroccans. From a total of 5.9 million SNVs detected, over 200,000 were not identified by 1000G and were not in the extensive gnomAD database. We summarise the SNVs by genomic position, type of sequence gene context and effect on proteins encoded by the sequence. Analysis of the overall genomic information of the Moroccan individuals to individuals from 1000G supports the Moroccan population being distinct from both sub-Saharan African and European populations.

**Conclusions:**

We conclude that Moroccan samples are genetically distinct and lie in the middle of the previously observed cline between populations of European and African ancestry. WGS of Moroccan individuals can identify a large number of novel SNVs and aid in functional characterisation of the genome.

## Background

The accepted model of human evolution is that *Homo sapiens* appeared in Africa around 200,000 years ago [1]. Adaptation to modern humans probably occurred gradually in several different locations [2]. Until recently, the oldest *H. sapiens* fossils were from Ethiopia, dated as 195,000 years old [3]. However, fossils discovered in Morocco have been re-dated as 300,000 years old [4]. There is evidence of modern humans in North Africa from about 160,000 years ago [5, 6]. Populations outside of Africa are thought to have all originated by serial colonisation from Africa [2] and still retains a greater genetic diversity within Africa than in any other region [7]. However, the majority of genome sequencing studies have largely excluded Northern African individuals [8]. Understanding this missing diversity can help to illuminate fundamental function within the human genome. Sequencing Moroccan individuals may reveal variants that can contribute to our understanding of human biology and disease.

A major application of genomics is understanding the causes of human disease. Nearly all diseases have a genetic component but every individual carries millions of variations from the reference genome and therefore pinpointing pathogenic variants is extremely challenging. Characterising the full spectrum of human genome variation in healthy individuals is a valuable step and necessarily this includes wide sampling of different populations. While many variations are seen in multiple populations, some are variations are restricted or very rare. The pattern of variation can also tell us about human evolution and migration.

Genetic analysis of mtDNA and Y-chromosomes showed that North African populations are mixtures of autochthonous and populations from the Middle East, Europe and sub-Saharan Africa [9]. The human history of North Africa is characterised by multiple migration movements, admixtures and founder effects. Recent genomic sequence of individuals from North African supports the theory of back-to-Africa migration in the pre-Holocene period ~ 12,000 years ago when the Berbers tribe migrated from Arabia [10]. This was followed by many population movements resulting from invasions and migrations by such groups as the Phoenicians, Romans, Vandals, Byzantines, Jew, Arab and more recently Spanish and French [11].

For many centuries, North African populations have been mistakenly viewed as continuous with sub-Saharan populations. However, Arredi et al. in 2004 showed that North African populations have predominantly Neolithic origin using Y-chromosome DNA markers [12]. Analysis of SNVs from 2099 individuals in 43 populations showed that North African populations are closer to European and Near East than they are to Sub-Saharan populations [7]. More recently, gene flow from North Africa enriches genetic diversity in southern Europe, which is not the case for Sub-Saharan populations. In fact, between 4 and 20% of Southwestern European genomes were assigned to North African ancestral cluster whereas only Sub-Saharan ancestry was detected at < 1% in Europe [7].

The North African population, which exceeds 160 million people, has not been represented in international genome sequence consortiums including the 1000 Genomes Project (1000G) and The African Genome Variation Project. To further characterise the North African genome and establish relationship with genomes from other ethnical groups we decided to perform whole genome sequencing (WGS) of three individuals from North Africa. We propose that the distinct genetics of North African’s should be a focus for characterising (dys) function within the human genome.

## Results

### Sequencing

Sequencing produced 487 million paired-end reads for the first sample and approximately half that number for the other two samples. Over 99.7% of reads mapped to the reference for all samples. The mean depth was 30X for the first sample and 16X for the other samples. The percentage coverage of the samples at 10X was 88-92%.

### Quality control

**Fig. 1:**
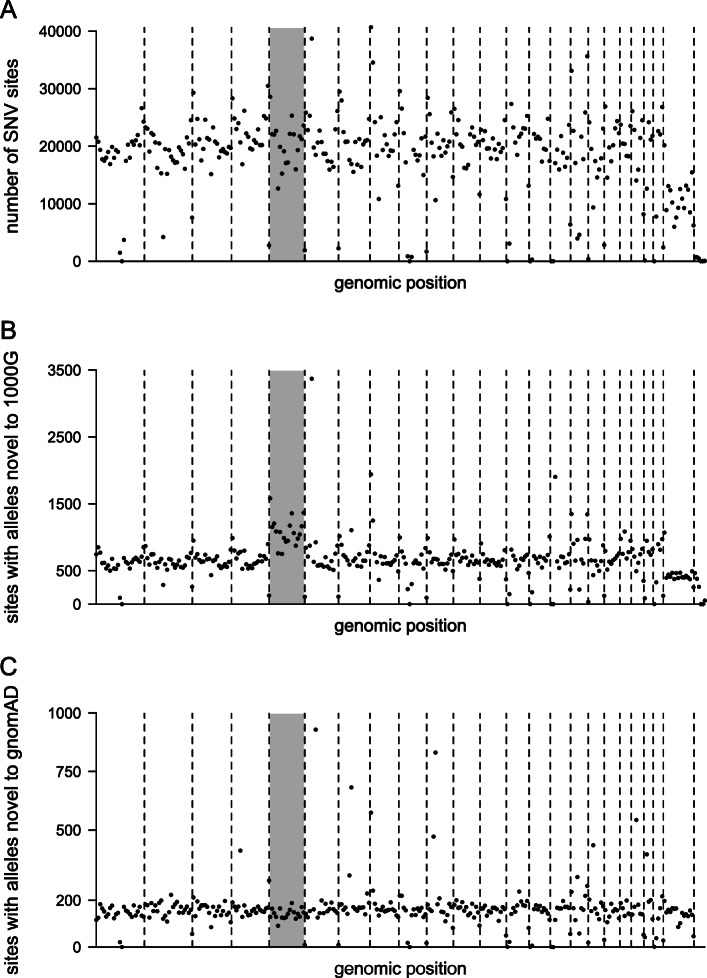
Distribution of SNV sites across the genome. Number of SNVs is shown for 10 Mb blocks of each chromosome. Lines indicate chromosome boundaries. Shaded area is chromosome 5. **a** All SNV sites identified in the Moroccan individuals. **b** SNV sites with alleles not found in 1000G. **c** SNV sites with alleles not found in gnomAD. The value for 30-40 Mb on chromosome 6 was highest, at 4094, but is omitted to better represent the majority of the genome. No values are shown for the Y chromosome because GnomAD did not make calls for this chromosome

To design quality control, the distribution of values for pairs of metrics was compared for true (matching alleles in HapMap phase 3 or the Omni 2.5 M SNP chip) and other SNV sites. The starting ratio of true to other sites was 0.72. The first filtering threshold applied was − 2.5 < MQRankSum< 1 and QD > 7 or QD > 0.5 and − 0.01 < MQRankSum< 0.01; this retained 99.61% of true sites and had a discrimination factor of 1.088. The second threshold was MQ > 50 or FS = 0 and MQ > 40; this retained 99.4% of remaining true sites and had a discrimination factor of 1.054. VariantRecalibration was then run on the remaining SNVs with DP, SOR, ReadPosRankSum and FS as input metrics. The option --maxIterations had to be increased to 500. The output graphs were greatly improved from the first attempt and satisfactory. GATK’s ApplyRecalibration was used to add VQSLOD scores to the VCF file. A threshold of VQSLOD>-0.5 was chosen. This retained 99.8% of remaining true sites with a discrimination factor of 1.024. The overall process retained 99.2% of true sites and increased the ratio of true to other sites by 17%; 9% of SNVs were removed. After quality control, there were 5,881,260 biallelic SNVs and 5359 multiallelic SNVs (Fig. 1a).

### A high proportion of Moroccan SNVs are novel and predicted to be functional

**Fig. 2:**
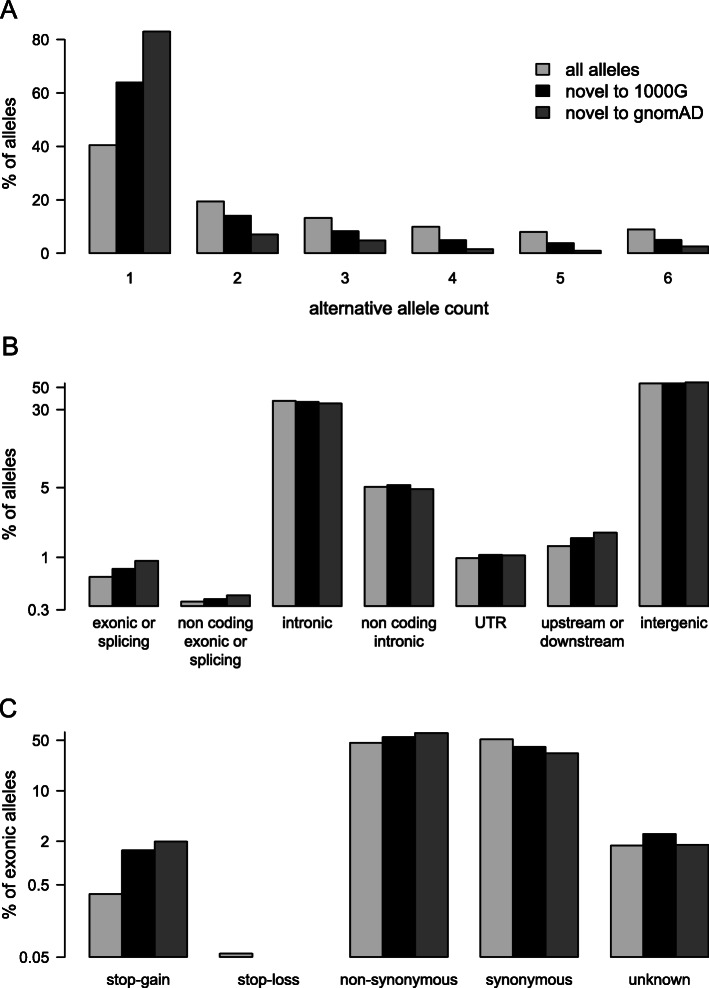
Properties of discovered SNVs. Results are shown for all SNV alleles, alleles not found in 1000G and alleles not found in gnomAD. Light grey bars are percentage of all alleles in each class, black are percentage of alleles novel to 1000G in each class, and dark grey are percentage of alleles novel to gnomAD in each class. **a** Distribution of number of copies of the alternative allele in the three Moroccan individuals. **b** Distribution of alleles across different types of sequence. **c** For exonic alleles, distribution of functional effects. For (**b**) and (**c**), the Y axis is log10 scaled and annotation was performed in ANNOVAR version 16-04-18 with the RefSeq gene model

There were 215,691 SNV sites (4769 multiallelic) that were not reported in 1000G. A further 3600 sites discovered in 1000G had a different allele in the Moroccan individuals. There was a concentration of novel SNVs on chromosome 5 and a very high number of novel SNVs between 30 and 40 Mb on chromosome 6 (Fig. 1b). In view of this surprising finding we checked whether it could be an artefact related to variant filtering by 1000G [13]; a similar comparison with gnomAD SNV sites (version 2.1.1) does not retain the peak on chromosome 6 suggesting that this result was indeed artefactual (Fig. 1c). Overall we identified 55,042 SNV sites that were not identified in gnomAD. Only 1% of SNV sites had missing genotypes. A small number, 0.6%, of the SNV alleles were located in exons or splice sites; interestingly novel SNVs were enriched in these regions (Fig. 2b). Moreover, 1.5% of novel exon SNVs were nonsense variants which is 4-fold higher the value for reference and novel alleles combined (Fig. 2c). Overall these observations are consistent with significant numbers of high impact novel variants within Moroccan individuals.

### Moroccan genomes form a distinct population

**Fig. 3:**
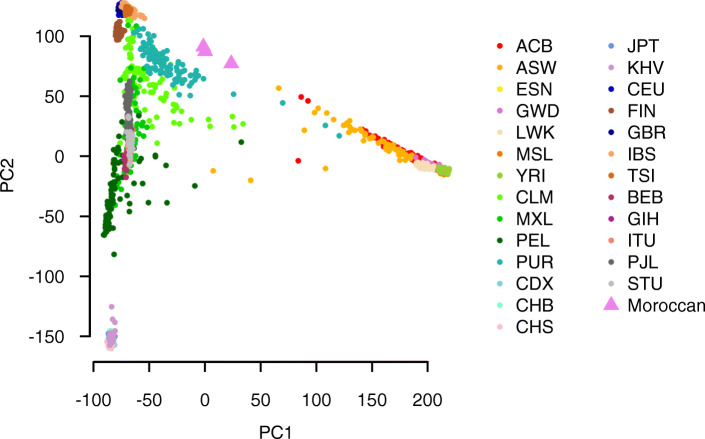
Inferred shared ancestry of Moroccan individuals to global populations. The first (PC1) and second (PC2) principle components of individuals from 1000G and the Moroccan individuals are shown. Principle components were calculated from the genotype matrix of high-quality, LD-pruned SNPs from 1000G. The Moroccan individuals were genotyped at the same sites and values predicted with the same model. Three letter codes in the legend are from 1000G. The continental areas of the populations are as follows: ACB, ASW, ESN, GWD, LWK, MSL and YRI - Africa; CLM, MXL, PEL and PUR - the Americas; CDX, CHB, CHS, JPT and KHV - East Asia; CEU, FIN, GBR, IBS and TSI - Europe; BEB, GIH, ITU, PJL, STU - South Asi

We then analysed the entire Moroccan genomes by principal components analysis to determine similarity to 1000G populations. Data projected onto PC1 and PC2 calculated from 1000G placed the Moroccans between European and African 1000G populations (Fig. 3).

## Discussion

Sequencing new populations is important to understand the diversity and ultimately function of the human genome. The Moroccan population has been neglected in part because of assumptions that it is not distinct and offers little new information. Our study demonstrates that this is not the case. In fact, Moroccans harbour a large number of novel SNVs and are genetically distinct from European and Sub-Saharan populations.

The study of pathogenic genetic variation rests upon successful filtering of likely benign variants. Large scale population studies such as gnomAD [14] have significantly advanced this effort by distinguishing common from rare variants; rare variants are more likely to be under negative selection and therefore pathogenic. The amount of new information per genome is maximised when individuals are drawn from different populations. Our study demonstrates that Moroccans carry a large number of high impact genomic variants in coding regions. None of the individuals in this study suffered a genetic disease at the age of sampling and therefore it is likely that much of the genetic variation we have described is benign and should be excluded in future attempts to define the genetic architecture of diseases.

A high number of novels SNVs were found in a region of chromosome 6 and on chromosome 5 however further analysis suggests that this may have been an artefact related to 1000G methodology. This is an important finding which may be useful for other similar population genetics work.

## Conclusions

WGS in three Moroccan individuals without significant pathology identified a relatively large number of novel SNVs particularly within conserved coding regions. We conclude that focused inclusion of Moroccan individuals in future population-level WGS studies is a cost-effective method to maximise the discovery and characterisation of human genetic variation.

## Methods

### Sample selection and sequencing

Volunteers from different Moroccan regions in the far West of Africa, were recruited in Specialised Hospital of Rabat. All participants were informed regarding the study and written consent was obtained from them according to research protocol presented and approved by local research committee of the Faculty of Medicine and Pharmacy at the University Mohammed V in Rabat. Blood samples were coded and DNA was extracted according to standard protocol. Three samples were randomly chosen for sequencing including one Arab speaker and two Berber speakers; a language was assigned based on the language spoken by all members of the preceding three generations. The samples were paired-end whole genome sequenced on an Illumina HiSeq 2500 with a 100 bp read length. The first sample was run on five lanes across three Rapid Runs. The other two samples were each run on a single lane in High-Output Mode. FASTQ files were produced for each lane and sample by bcl2fastq (version 1.8.4) with adapter trimming and allowing a single mismatch in the index sequence.

### Variant calling

Reads were aligned to the build 37 version of the human reference genome with decoy sequences that was used in phase 3 of 1000G, using the MEM algorithm in BWA (version 0.7.15a). Duplicate reads were marked by Picard (version 2.8.0). For the first sample, alignment and duplicate marking was performed per lane; the data were then merged into a single BAM file with Picard and duplicate marking repeated. Base quality scores were adjusted by BQSR from GATK (version 3.7). Alignment statistics were generated with flagstat from SAMtools (version 1.3.1) and depth and coverage statistics were produced using GATK’s DepthOfCoverage restricted to the autosomes and with a minimum base quality of 10 and minimum mapping quality of 20. A gVCF file of all sites was generated for each sample by GATK’s HaplotypeCaller with the options --emitRefConfidence BP_RESOLUTION. One VCF file of all sites with evidence of a difference from the reference in any sample was produced from the three gVCFs with GATK’s GenotypeGVCFs.

### Quality control

Sites in the VCF file were excluded if the metric QD was absent, the metric DP (in the sample data) was zero for all samples, or the metric AD was zero for all alternative alleles in all samples. Sites outside the autosomes were excluded. The intention was to perform variant quality control using GATK’s VQSR. VQSR models a new site metric (VQSLOD) from selected metrics by machine learning on the dataset. The algorithm trains on a subset of sites that are defined as true sites (sites in the dataset that are in HapMap phase 3 or the Omni 2.5 M SNP chip). VariantRecalibrator was run in SNP mode with QD, MQ, MQRankSum, ReadPosRankSum, FS, SOR and DP as input metrics. However, VQSR output graphs suggested poor performance perhaps due to the small sample size; the probability density function (PDF) was not smooth, with low and high values interspersed, and no clear pattern across the parameter space; and the frequency distributions of MQ and MQRankSum in the true sites had a sharp spike, which may have affected the model fitting because VQSR assumes metrics are normally distributed. We therefore investigated first excluding sites by threshold values for MQ and MQRankSum and then running VariantRecalibrator without those metrics. Quality control was only developed for single nucleotide variants (SNVs). Given the difficulties of quality control for SNVs and the higher error rate for calling insertions or deletions (INDELs), all further analysis was with SNVs only.

To choose threshold values MQ and MQRankSum, the distribution of values in the VCF file for pairs of the seven metrics were examined using R (version 3.3.1). Metrics were inspected in pairs so the joint distribution across the values of two metrics could be seen. Adopting the technique of a set of true sites, SNV sites were classified as either true or other. True sites had alleles matching SNPs in HapMap phase 3 or the Omni 2.5 M SNP chip. Frequency distributions were compared for true and other sites to identify ranges of values that were seen at other sites but were rare at true sites. Different thresholds were evaluated by the percentage of true sites kept and the ratio of this to the percentage of other sites kept. This ratio will be described as the discrimination factor because it is the factor by which the ratio of true to other sites is increased by filtering.

### Identification of novel SNVs

The quality controlled SNVs were compared to the SNVs from phase 3 of 1000G (version 5a, [15]). 1000G discovered over 80 million SNVs from whole genome sequencing 2535 individuals. The 1000G VCF file includes multiallelic sites where alleles are written in a complex way to enable SNVs and indels to be represented at the same site. A custom Perl script was used to separate alleles and convert them to standard notation and left align INDELs. SNV alleles in the Moroccan individuals that were not reported in 1000G were determined. The distribution of SNV sites and sites with alleles not in 1000G across the genome was explored by dividing each chromosome into 10 Mb blocks. Custom Perl scripts were written for this and other analyses.

SNVs were also compared to SNVs in the Genome Aggregation Database (gnomAD, [14] version 2.1.1), genomes data. The data in gnomAD are an amalgamation from multiple large-scale sequencing studies, primarily disease case-control studies, totalling 15,708 genomes. Individuals with severe childhood-onset diseases and their first-degree relatives were removed and a set of unrelated samples were selected for gnomAD. The gnomAD variant set was generated by reanalysing the original FASTQ files through a single pipeline followed by joint variant-calling. GnomAD provides all called variants with flags for those that failed quality control. To reduce the chance of novel SNVs being false positives, we determined alleles that were not in gnomAD and alleles that were called by gnomAD but filtered out for quality were excluded. GnomAD genomes does not have Y chromosome variants so these could not be compared. The distribution of SNV sites with novel alleles not in gnomAD was assessed in the same way as for alleles not in 1000G.

### SNV characterisation

The number of copies of the non-reference alleles in the Moroccan individuals was counted. SNV sites where there was no genotype call for one or more individuals were excluded. The allele count spectrum was also evaluated for SNVs novel to 1000G and SNVs novel to gnomAD.

The filtered VCF file was converted into the input format for ANNOVAR (version 16-04-2018) including rewriting all multiallelic sites so that each allele was on a separate line, with a custom Perl script. RefSeq (version x) gene annotations were added with ANNOVAR’s annotate_variation.pl program and the option -separate, which provides annotations for all alternative transcripts. The number of alleles in different types of sequence was determined from the variant_function output file. Each allele was allocated to only one type; precedence for multiple annotations was, from high to low, exonic or splicing (within the first or last two bases of an intron), ncRNA_exonic (non-coding RNA exonic) or ncRNA_splicing, UTR5 or UTR3, intronic, ncRNA_intronic, upstream or downstream, intergenic. For exonic alleles, the number with different functional effects was determined from the exonic_variant_function output file. Each allele was allocated to only one effect; the precedence for multiple annotations was stop-gain, stop-loss, non-synonymous SNV, synonymous SNV or unknown. The distribution of SNV alleles across sequence type and functional effects was also measured for alleles novel to 1000G and alleles novel to gnomAD.

### Inference of Moroccan ancestry from genome-wide pattern of variation

The Moroccan samples were projected onto the first two principal components from a principal component analysis (PCA) of 1000G samples. Phase 3 (version 5a) of 1000G was used. A high quality, representative set of SNVs was selected as follows. Biallelic sites on the autosomes with a minor allele frequency > 5% were extracted. Any that were within 5 bp of another SNV or INDEL were excluded. The genotypes for these SNVs were loaded into PLINK/SEQ (version 0.10) and only SNVs within the 1000G phase 3 ‘Strict’ mask that did not deviate from Hardy-Weinberg equilibruim (*P* value≥10-6) were retained. Genotypes at these SNVs were recalled for the Moroccan samples with GATK’s GenotypeGVCFs with the options -L, −ip 100 and --includeNonVariantSites as nearly a third of the sites were not in the original VCF file. A small number (0.1%) of sites were still not called and were excluded. The genotypes were unchanged for sites that were in the original VCF file. Sites that had been excluded in the Moroccan samples for quality control, were within 5 bp of another variant in the Moroccan data or had a different alternative allele from 1000G were removed. SNVs were also filtered based on site and sample metrics in the Moroccan data; sites that were homozygote reference for all samples were kept if they had QUAL≥30, and DP ≥ 10 and RGQ ≥ 20 for all samples; variant sites were kept if they had DP ≥ 10 and GQ ≥ 20 for all samples. Filtering based on the Moroccan data excluded 22% of the selected 1000G sites.

The 1000G genotypes for the high-quality sites were loaded into PLINK (version 1.07). SNVs within two regions of extended LD (chr6:25,000,000-35,000,000 and chr8:7,000,000-14,000,000) were removed then LD pruning was performed with the options --indep 50 5 2. This resulted in 310,000 SNVs for PCA. The 1000G genotypes were replaced with indicator variables 0, 1, 2, equal to the number of alternative alleles present. Principle components were calculated in R by the prcomp function with options centre = TRUE and scale = T. Principle component values for the Moroccan individuals were calculated from the 1000G model with the predict function.

Availability of data and materials: all data are available at http://www.ncbi.nlm.nih.gov/bioproject/660888; BioProject accession number for the submission: PRJNA660888; BioSample accessions SAMN15963187, SAMN15963188, SAMN15963189.

## Data Availability

Data will be made available through the University of Sheffield Data Repository (https://www.sheffield.ac.uk/library/rdm/orda).

## Abbreviations

SNV: Single nucleotide variant
WGS: Whole genome sequencing
1000G: 1000 Genomes Project
gnomAD: Genome Aggregation Database
PCA: Principal component analysis
GATK annotations: Genome Analysis Tool Kit
MQRankSum: Mapping Quality Rank Sum Test
QD: Quality by Depth
MQ: Root Mean Square Mapping Quality
FS: Fisher Strand
ReadPosRankSum: Read Position Rank Sum Test
VQSR: Variant Quality Score Recalibration
VQSLOD: Variant quality score log-odds
SOR: Strand Odds Ratio
